# Integrated Management Strategies for Blackleg Disease of Canola Amidst Climate Change Challenges

**DOI:** 10.3390/jof11070514

**Published:** 2025-07-09

**Authors:** Khizar Razzaq, Luis E. Del Río Mendoza, Bita Babakhani, Abdolbaset Azizi, Hasnain Razzaq, Mahfuz Rahman

**Affiliations:** 1 Department of Plant Pathology, North Dakota State University, Fargo, ND 58102, USAbita.babakhani@ndsu.edu (B.B.); abdolbaset.azizi@ndsu.edu (A.A.); 2 Department of Plant Pathology, University of Agriculture Faisalabad, Faisalabad 38000, Punjab, Pakistan; hasnainrazzaq476@gmail.com; 3 WVUExtension Service, West Virginia University, Morgantown, WV 26506, USA; mm.rahman@mail.wvu.edu

**Keywords:** climate change, integrated disease management, blackleg, canola, crop rotation, durable resistance

## Abstract

Blackleg caused by a hemi-biotrophic fungus *Plenodomus lingam* (syn. *Leptosphaeria maculans*) poses a significant threat to global canola production. Changing climatic conditions further exacerbate the intensity and prevalence of blackleg epidemics. Shifts in temperature, humidity, and precipitation patterns can enhance pathogen virulence and disease spread. This review synthesizes the knowledge on integrated disease management (IDM) approaches for blackleg, including crop rotation, resistant cultivars, and chemical and biological controls, with an emphasis on advanced strategies such as disease forecasting models, remote sensing, and climate-adapted breeding. Notably, bibliometric analysis reveals an increasing research focus on the intersection of blackleg, climate change, and sustainable disease management. However, critical research gaps remain, which include the lack of region-specific forecasting models, the limited availability of effective biological control agents, and underexplored socio-economic factors limiting farmer adoption of IDM. Additionally, the review identifies an urgent need for policy support and investment in breeding programs using emerging tools like AI-driven decision support systems, CRISPR/Cas9, and gene stacking to optimize fungicide use and resistance deployment. Overall, this review highlights the importance of coordinated, multidisciplinary efforts, integrating plant pathology, breeding, climate modeling, and socio-economic analysis to develop climate-resilient, locally adapted, and economically viable IDM strategies for sustainable canola production.

## 1. Introduction

**Figure 1:**
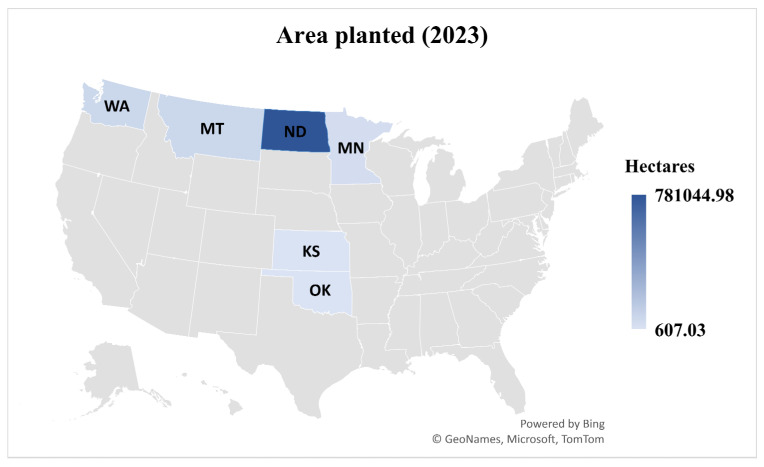
Estimated hectarage planted with canola in 2023 in the top six producing US states, ordered from smallest to largest: Kansas (607.03), Oklahoma (1,214.06), Minnesota (32,374.88), Montana (66,772.19), Washington (66,772.19), and North Dakota (781,044.98). (Source: https://www.uscanola.com/crop-production/spring-and-winter-canola, accessed on 9 September 2024).

**Figure 2:**
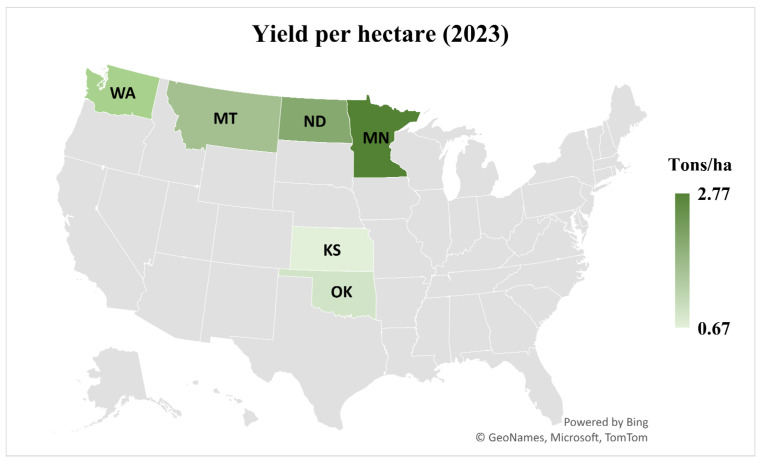
Canola yield per hectare for the 2023 growing season ranged from 0.27 to 1.12 tons per ha (600–2470 lb per acre) in the top six canola-producing states. Minnesota recorded 2.77 tons/ha, followed by North Dakota (2.03), Washington (1.84), Montana (1.59), Oklahoma (0.9), and Kansas (0.67). (Source: https://www.uscanola.com/crop-production/spring-and-winter-canola, accessed on 9 September 2024).

Blackleg disease of canola, caused by the hemi-biotrophic fungus *Plenodomus lingam*, formerly known as *Leptosphaeria maculans*, is a significant threat to the canola industry of North Dakota. This state contributes more than 85 percent of USA canola production [1]. The latest data on canola planted area, harvested area, and yield per acre across top canola-growing states of the USA during the 2023 growing season are given in Figure 1 and Figure 2 (source: https://www.uscanola.com/crop-production/spring-and-winter-canola; cited on 9 September 2024). Blackleg is endemic in all North Dakota production areas [2]. It can cause up to 20% yield losses annually. However, under favorable conditions, disease severity can rise exponentially to cause up to 99% yield losses [3,4].

Yield loss due to disease is influenced by environmental conditions and the growth stage at which plants are initially infected. Initial fungal infections of leaves can be caused by ascospores or pycnidiospores under favorable environmental conditions. Upon germination on the leaf surface, hyphae penetrate the epidermis via stomata or wounds caused by mechanical damage or insect feeding [5]. Inside the epidermis, *P. lingam* grows biotrophically with an initial establishment phase followed by an exploration phase that helps the fungus to spread through intercellular spaces [6]. During this phase, the fungus releases enzymes that trigger host cell death and form lesions [7]. This phase continues until the production of pycnidia that release the spores to spread the infection to nearby plants through rain splashes [8]. At the same time, the necrotrophic phase initiates in areas surrounding the point of penetration. The hyphal growth continues towards the main stem in the biotrophic mode, where the hyphae primarily travel through the phloem. The necrotrophic phase in the stem leads to stem cankering, which is the most damaging phase of the disease. The fungus overwinters in stubbles/plant debris, where it produces pseudothecia. Mature pseudothecia release ascospores in the spring that are carried and spread by wind to serve as primary inocula [9]. Typical blackleg symptoms include brown leaf lesions with dark borders, often caused by pycnidiospores (secondary infections). These lesions may or may not have a yellow halo around them, depending on the production of toxins by some isolates. Blackleg can also lead to stem cankers, resulting from initial infections of seedling tissues in the crown area. Additionally, late infections can cause superficial cankers on the upper portions of the stem and branches, leading to stem cracks, root rot, stunted growth, and pod infection [10,11].

Climate change poses a significant threat to the agriculture sector. Global temperatures rose about 1 °C (1.8 °F) from 1901 to 2020, leading to various anomalies in cellular life forms [12]. Environmental conditions like temperature and moisture influence fungal infections on plant surfaces. More specifically, leaf wetness duration under a favorable temperature determines fungal spore germination and successful infection [13]. Higher precipitation and relative humidity can potentially increase leaf wetness duration. For example, from 1850–1900 to 2010–2019 global temperatures increased by 0.8 °C to 1.3 °C (1.4 to 2.3 °F) [14], with a significant increase in precipitation [15]. Multiple studies on temperature and moisture have demonstrated that both factors significantly influence the severity and progression of blackleg disease in canola, providing important insights into the impact of climatic changes on disease management. Metabolomic analyses revealed that in resistant canola cultivars elevated temperatures (28 °C/82 °F) can suppress defense-related pathways, including amino acid and lipid metabolism, and compromise resistance effectiveness [16]. Further, another study showed that thermal time and soil moisture accounted for a large portion of the variability in the reaction to blackleg disease of resistant and susceptible cultivars in Australia [17]. Furthermore, wetness period durations and temperature can have a large impact on blackleg infection. Leaf lesions developed on plants incubated at 15 to 18 °C (59 to 64 °F) with 96 h of prolonged leaf wetness, while stem infections were greater at 20 to 23 °C (68 to 73 °F) with only 48–72 h of wetness, and no stem lesions developed at 6 to 8 °C (43 to 46 °F), even after 42 days of incubation post-inoculation [13]. Canola-growing regions with frequent rainfall and warm humid conditions are more affected by blackleg. In North Dakota temperatures ranging between 13 and 21 °C (55 to 70 °F), with relative humidity above 80%, result in maximum spore release, which facilitates primary and secondary infections with blackleg disease (NDSU Extension, PP1988 (https://www.ndsu.edu/agriculture/sites/default/files/2022-11/pp1988.pdf; accessed on 26 June 2025). As climate changes are accentuated in the region, these observations underscore the critical need for rigorous field-based research that could result in improved disease forecasting models and integrated management strategies.

Climate change has been affecting seasonal warming and cooling cycles, affecting crops with both heat and freezing stress. In recent years, sudden rises in temperatures in early spring have pushed blooms to open earlier than usual at a time when the probability of frost occurrence remains active. Thus, frost events have been causing crop failure, especially in fruit trees, by killing blooms in recent years. Spring frosts have been affecting the growth and health of plants in Europe and North America more often. For example, freezing temperatures and frost caused severe damage to crops, including oilseed rape, potatoes, and sugar beets, during April 2021 in Europe [18]. Under cold weather conditions, the blackleg fungus tends to survive by forming pseudothecia, the fruiting bodies of the fungus, which can endure winter frosts, although their ability to release ascospores may be reduced. Despite this, the ascospores within pseudothecia remain viable and capable of germination [19]. On the other hand, warmer temperatures increase the growth and sporulation of the fungus that causes blackleg disease in canola [20]. Warmer winters and higher precipitation levels in recent years in North Dakota created disease-favorable conditions for *P. lingam*, an indication of the impact of climate on disease incidence and severity [21,22]. A few past studies showed a significant rise in blackleg incidence, severity, disease pressure, and geographical spread [23,24]. To mitigate the effect of climatic changes on disease prevalence and severity, effective management strategies must be developed, specifically those that incorporate quantitative and qualitative resistance genes and advanced disease forecasting systems and optimize fungicide application [25,26,27]. Integrated disease management (IDM) combines these approaches with cultural and biological control methods [28]. For example, stubble management, crop isolation, and three to four years of crop rotation can be key to minimizing inoculum production and disease pressure [29]. Likewise, resistant varieties of canola developed through breeding programs that mainly focus on major R-genes and quantitative resistance are an important component of IDM strategies [30]. The durability of qualitative resistance against *P. lingam* is enhanced when combined with quantitative resistance [31]. Crop rotation and the use of fungicides can be critical in managing blackleg of canola. For example, timely application of fungicides can protect canola crops during the vulnerable growth stage against *P. lingam* [32], and rotation of canola with non-host crops can reduce the inoculum of blackleg [33]. Implementing IDM strategies not only helps in reducing disease incidence and severity but is also environmentally sustainable.

**Figure 3:**
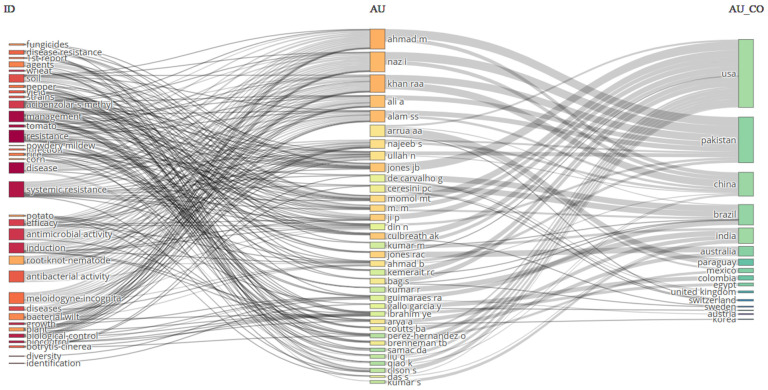
Comprehensive bibliometric analysis of studies published between 2001 and 2025 that were conducted on integrated disease management strategies adopted for different crops worldwide. The colors represent thematic groups for research topics (e.g., orange for pathogens, red/purple for resistance, pink for chemicals), author affiliations by topic intensity (yellow to orange shades in the center), and countries by author contribution frequency (green shades on the right).

**Figure 4:**
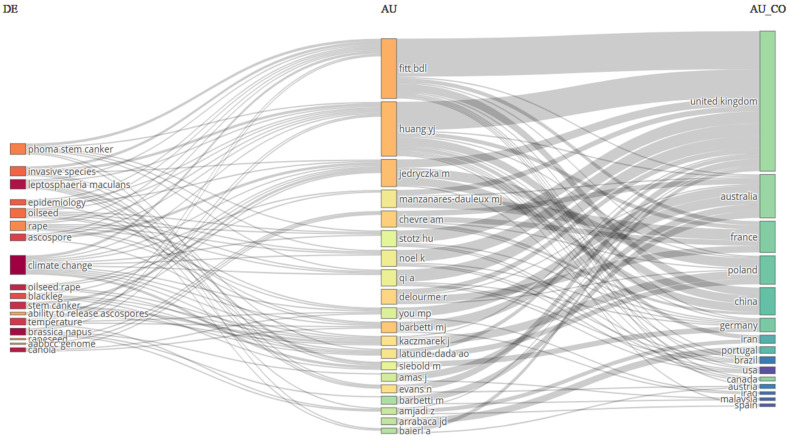
Bibliometric analysis of studies published between 2001 and 2025 that were conducted on climate change and blackleg of canola.

A hallmark of this review is the analysis of published work on IDM to show the promising trend of the system in sustainable crop production methods. A comprehensive overview of work conducted on various crops using IDM across different parts of the world is presented through a bibliometric analysis, represented in Figure 3. The figure includes three fields, countries on the right side, authors in the middle, and major keywords on the left side, and shows the relationship between the authors of different countries leading with publications in various journals across the world. The USA leads in terms of publications, followed by Pakistan, China, Brazil, India, and Australia. Overall, the figure identifies the global research trends, collaborations, and focus on IDM strategies that can be adopted for specific crops. Further, the bibliometric analysis of the effect of climate change on blackleg of canola (Figure 4) shows that research on blackleg of canola in the context of climatic variability has been concentrated in certain regions. The connections between different fields explain the thematic and geographical focus of this niche, but the critical research domains are led by the United Kingdom, Australia, France, Poland, and China. This analysis identifies the key contributors to IDM globally and highlights the underrepresented regions where more research is needed. This review further examines the application of IDM strategies to mitigate the impact of climate change on blackleg incidence and severity in canola. By synthesizing recent research and integrating diverse management approaches, it provides a comprehensive understanding of IDM strategies to address climate-induced challenges through bibliometric analysis, supporting the sustainability of canola production and thus contributing to global food security.

## 2. Survival Structures and Infection Biology of Blackleg Pathogen in Canola

*Plenodomus lingam* persists on infected plant debris through its sexual structures known as pseudothecia and its asexual structures called pycnidia. The pseudothecia produce ascospores, while pycnidia generate pycnidiospores. Both types of spores act as primary sources for initiating blackleg infections [33,34]. Optimal temperatures for the maturation of pseudothecia range from 15 to 20 °C (59 to 68 °F) [35]. Additionally, pseudothecia development is enhanced by conditions of high moisture, such as regular rainfall or elevated humidity levels [36]. Favorable environmental conditions characterized by substantial rainfall (exceeding 1 mm), moderate–high relative humidity (above 80%), and temperatures between 13 °C and 18 °C (55 and 64 °F) are known to stimulate spore liberation [37]. The maturation period for pseudothecia differs across various regions and seasons and is influenced by environmental conditions such as temperature, rainfall, and relative humidity levels. While ascospore production may take place during the entire growth period, the occurrence of conducive weather conditions at the cotyledon and seedling stages of canola is particularly important, as they can determine whether economically significant losses will occur. The impact of temperature, rainfall, and relative humidity on the development of *P. lingam* pseudothecia, the fruiting bodies that produce ascospores, has been studied in Australia, Canada, Europe, and the USA (North Dakota) [34,37]. In a study by Khangura et al. [37], it was observed that pseudothecia took between 73 and 192 days to mature in Australia, whereas the maturation process required only 16 to 51 days in France [36]. Ascospores began to form on canola stubble one month after harvest in Ontario, Canada [38]. Conversely, the onset of a harsh winter period after the canola harvest necessitated a longer span of several months for ascospore production in Saskatchewan, Canada. Toscano-Underwood et al. [35] found that the maturation of pseudothecia was expedited at a temperature of 20 °C (68 °F) in England compared to 5 °C (41 °F) in a regulated environment. Similar findings were reported by Petrie in Canada [39], where a 15 °C (59 °F) temperature was conducive to the production of ascospores in naturally infected debris. In France, a consistent average temperature of 14 °C (57 °F) coupled with 2.5 mm of rainfall every 3 to 4 days created optimal conditions for the maturation of pseudothecia. Additionally, environments with no rainfall but periods of almost 100% relative humidity fulfilled the moisture needs for the process. Furthermore, Salam et al. [40] observed that a period of 43 suitable days characterized by a 10-day average temperature below 22 °C (72 °F) and a minimum weekly rainfall of 4 mm is essential to produce ascospores following the harvest period in Western Australia. Petrie [41] and Wang [42] highlighted that rainfall at regular intervals is more critical than the cumulative rainfall amount for the generation of ascospores. However, ascospore dispersal was irregular under elevated temperatures and diminished moisture levels. In another study, Petrie [39] documented that 15 °C (59 °F) was the optimal temperature for ascospore production on stubble that was moistened with water two to three times daily. Conversely, stubble that received only a daily water spray did not exhibit any pseudothecia development, indicating the need of moisture for the process. In North Dakota, analyses using logistic regression have shown a link between the maturation of pseudothecia and climatic factors such as air temperature, measured in cumulative heat units, and relative humidity, quantified by the number of hours when the RH exceeded 75% [43]. The same study showed that pseudothecia appeared sooner and reached maturity at a notably quicker rate when the temperature was at 18 °C (64 °F) compared to cooler conditions.

Production and maturation of pseudothecia is also influenced by stem moisture conditions. Precipitation regimes that keep stem residues moist, like three to five precipitation events of at least 2.5 mm spaced three to eight days apart in a period of 15–24 days with an average air temperature of 14 °C (57 °F), provide optimum conditions for pseudothecia maturation. Under these conditions, pseudothecia mature within 13–23 days. As air temperatures increase, precipitation events at shorter intervals are needed. For example, ten precipitation events of 2.5 mm at three-day intervals are optimum, but at cooler air temperatures, e.g., at 7 °C (45 °F), at least five precipitation events of 2.5 mm every four days are required. Longer intervals between precipitation events at 7 °C (45 °F) or 20 °C (68 °F) prevent pseudothecia maturation [44]. In the absence of precipitation, constant high air relative humidity (e.g., ≥95%) can promote maturation at temperatures ranging between 14 (57 °F) and 18 °C (64 °F) [44]. Alternatively, maintaining the soil moisture close to field capacity also promotes maturation, provided the temperatures are between 14 (57 °F) and 18 °C (64 °F) when stubbles are laid in the soil [43]. Upon reaching maturity, ascospores are released in response to conducive weather patterns. It has been observed that the occurrence of ascospore release in principal canola-producing areas globally coincides with the seasonal rainfall [45].

The pattern of seasonal and daily ascospore discharges is more strongly influenced by moisture, e.g., precipitation and relative humidity, than temperature. A study conducted in England and Poland [46], where winter canola is grown, showed that ascospore release occurred at any temperature between 5 (41 °F) and 20 °C (68 °F), while increasing amounts of precipitation in August and September resulted in an earlier release of ascospores. In Canada and the United States, where spring canola is grown, ascospores are detected in the air from mid-July until early August [36], while in western Australia, ascospores are detected in the air between May and June. In France, the decline in temperatures following summer and the accumulated precipitation facilitated the liberation of ascospores from pseudothecia [45]. As pseudothecia mature earlier under warmer and wetter conditions, ascospores are expected to be more abundant and are likely to be discharged earlier in the season. These conditions may also result in prolonged ascospore showers, extending the period during which the pathogen can infect susceptible hosts. In Canada, ascospores and pycnidiospores are released mainly between 9 pm and 4 am, coinciding with relative humidity levels exceeding 80% and temperatures fluctuating between 13 (55 °F) and 18 °C (64 °F) [36].

Ascospores are primarily spread by wind. Factors such as the wind direction and speed, the structure of the crop canopy, and the landscape’s contours influence how far an ascospore can travel [45,46]. Ascospores of the *P. lingam* species can traverse considerable distances and stay alive for as long as one month under dry conditions, with temperatures spanning from 5 (41 °F) to 20 °C (68 °F) [45]. Due to its capacity for extensive dispersal, it is advised in Australia to maintain a field separation of at least 500 m from a source of inoculum [47]. Pycnidiospores are extruded from pycnidia and typically dispersed by rain splash [48]. Rainfall events ≥2 mm trigger pycnidiospores’ dispersal [46], whereas ascospores are typically released three days after rainfall [36]. Monitoring ascospore or pseudothecium production can lead to an appropriate timing of management strategies.

## 3. Current IDM Strategies for Blackleg of Canola

**Figure 5:**
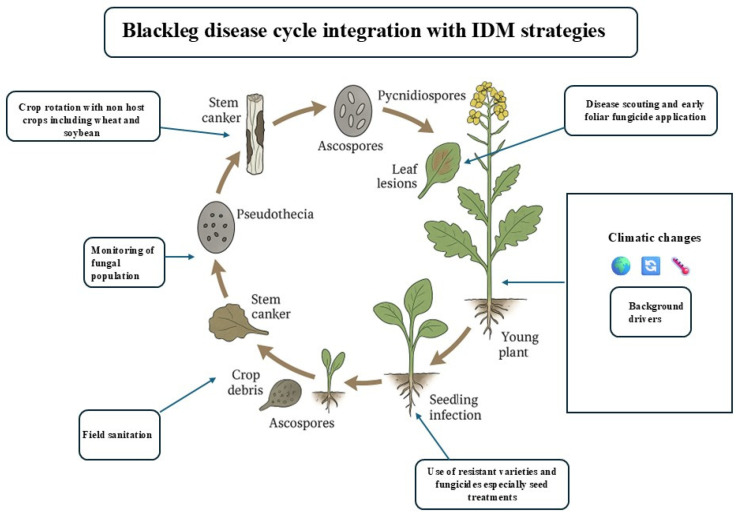
A schematic diagram recreated to emphasize integrated disease management (IDM) strategies and their integration with the blackleg disease life cycle under climatic changes.

**Figure 6:**
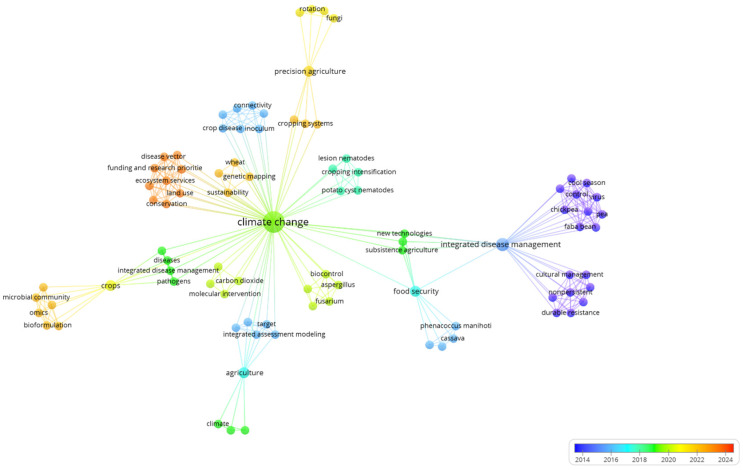
Bibliometric analysis of climate change and integrated disease management.

The ongoing climatic shift challenges the current management strategies for blackleg of canola. The best way to control this disease is through integrated disease management strategies that can mitigate the changing environmental effects on the crop. For example, the use of resistant germplasm, crop rotation [36], approved fungicide applications, and avoidance techniques, including cultural practices and biological control methods, are the best possible means to control canola blackleg [49]. For example, Zhang et al. [50] highlights the IDM strategies that can be adopted to break down the cycle of blackleg. Figure 5 highlights the key intervention points and their alignment with different stages of pathogen development and the co-occurrence map presented in Figure 6 reflects keyword relationships for climate change and integrated disease management (IDM) between 2014 and 2024. The network shows four major clusters, each representing distinct thematic areas, Climate Change (green clusters), IDM Strategies (purple clusters), Emerging Innovations (yellow/orange clusters), and Underrepresentation of Digital Technologies. The most prominent and densely connected node is climate change, which was strongly linked with sustainability, agriculture, and food security, highlighting climate change as a driver of broader ecological and agricultural changes. The purple clusters highlight the term integrated disease management (IDM) in the context of cool-season crops. This reflects continued emphasis on traditional, field-level IDM practices. However, the limited connectivity of these keywords with newer technological terms suggests a lack of integration with modern tools. The orange and yellow clusters show bioformulation, microbial community, and precision agriculture and increasing interest in biological control and data-enabled disease management, but the smaller node sizes imply that while these areas are growing, they are not yet central to mainstream IDM research. Digital tools represented by terms such as remote sensing, machine learning, and deep learning are either absent or weakly connected, highlighting a critical research gap. The integration of IDM strategies with the disease cycle of blackleg is depicted in (Figure 5), which emphasizes the need for IDM strategies to break down the life cycle of blackleg. To destroy the source of inoculum and keep the inoculum pressure low, crop stubble management and crop isolation can be key factors in canola blackleg management [36].

## 4. Methodology of Bibliometric Analysis

To investigate the evolving research landscape globally, a bibliometric analysis was conducted using VOSviewer (version 1.6.20). Two primary datasets, Web of Science and PubMed, were compiled to assess the scientific output.

The first Web of Science search targeted IDM strategies across major crops. The core research keywords were (“integrated disease management” or “IDM strategy”) and (rice, wheat, maize, corn, soybean, potato, tomato, cotton, barley, sorghum) and (“plant disease” or “crop disease” or “fungal” or “bacterial” or “viral”). This initial search yielded 235 articles, which were narrowed to 194 by selecting research articles only. Further refinement to focus on studies relevant to globally important crops reduced the dataset to 116 articles published between 2001 and 2025. Every article title was manually reviewed to ensure that it contained at least one of the relevant keywords, thereby confirming the article’s relevance to the research scope. RStudio version (2025. 05. 01) was used to conduct bibliometric analysis using biblioshiney R package. Three field plots were selected to identify global research trends and collaborations. The second dataset was retrieved from the Web of Science Core Collection and focused on climate change and blackleg disease in canola. The search string used was (“canola” or “Brassica napus”) and (“climate change” or “climate variability” or “global warming”) and (“plant disease” or “pathogen” or “disease resistance” or “fungal disease” or “blackleg” or “*Leptosphaeria maculans*” or “*Plenodomus lingam*”). This search returned a total of 96 articles from 2001 to 2025. After filtering to include only research articles, the dataset was reduced to 33 articles. The same analytic method was used for this dataset. Additionally, two PubMed searches were conducted to capture emerging trends in climate-responsive agricultural technologies. The first search used the keywords “climate change” and “integrated disease management,” while the second included “remote sensing,” “precision agriculture,” “deep learning,” “machine learning,” “crop detection,” and “hyperspectral imaging.” Results from both searches were filtered to include research articles, review articles, and conference papers published between 2014 and 2024. The final PubMed datasets were exported in CSV format for analysis. The search database was used for its robust metadata structure and seamless integration with bibliometric tools such as VOSviewer, enabling effective visualization and analysis of publication trends. All datasets were imported into VOSviewer for co-occurrence analysis using author keywords as the units of analysis. A minimum threshold of five keyword occurrences was applied to ensure relevance, and similar or synonymous terms were standardized using a thesaurus file to avoid redundancy. VOSviewer’s clustering algorithm was used to identify thematic groups, with each cluster visually represented by a distinct color. These analyses helped identify major research themes, collaborations, and the integration of emerging technologies within the context of IDM and climate change.

## 5. Host Resistance Breeding and Its Effectiveness Against Changing Climatic Conditions

The use of resistant germplasm has significantly protected canola crops from blackleg [50]. Many crops in temperate regions are expected to face environmental conditions like those found in different geographic zones due to global climatic changes [51], and the phenomenon is known as climate analogue. For example, a 1–2 °C increase in temperature would extend frost periods by 10–20 days and cause a 5–10% rise in summer precipitation in the Canadian Prairies by mid-century [52]. The whole phenomenon has implications for disease management in canola, and not all of them are bad. For instance, blackleg-resistant canola cultivars developed for warmer conditions could be deployed in cooler regions facing increasingly warmer environments [20]. Although resistant germplasm is efficient in controlling the blackleg pathogen, the continuous deployment of cultivars with single resistance genes imposes strong selection pressures on pathogen populations that can lead to the buildup of races capable of overcoming the resistance of the host [25] or cause Repeat-Induced Point (RIP) mutations [53]. In canola, the continuous use of germplasm carrying the blackleg resistance gene *Rlm1* resulted in a decrease in *AvrLm1*-carrying isolates in the pathogen population across the USA [54,55]. In other countries, like Australia, resistance derived from *B. rapa* subsp. *sylvestris*, which has a single dominant gene against blackleg, was overcome by the pathogen within three years of the release of commercial cultivars [56]. In Canada, resistance to blackleg was introduced in the early 1900s by replacing the susceptible cv. Westar with Quantum, Hi-Q, and High-Q2, which carried the resistance gene *Rlm3* [57]. While *Rlm3* is still present in most Canadian canola cultivars, it is no longer effective, as its corresponding avirulence gene, *AvrLm3*, is present in low frequencies in the pathogen population [52]. A review paper written by Rimmer [58] indicated that 12 resistance genes had been identified. A more recent review [59] indicates that at least four additional resistance genes have been identified; of these, three have been cloned. The genes *Rlm1*, *Rlm2*, *Rlm3*, *Rlm4*, *Rlm7*, and *Rlm9*, were detected in *B. napus* and have been mapped to linkage groups N7 and N10 [60,61,62,63,64], and the genes *Rlm8 and Rlm11* were detected in *B. rapa* [65,66], while *Rlm5* and *Rlm6* were detected in *B. juncea* [67], *Rlm10* was detected in *B. nigra* [68], and *LepR1*, *LepR2*, *LepR3*, *LepR4*, and *RlmS* were detected in *B. rapa* subsp. *sylvestris* [69,70,71,72]. Two other genes, *BLMR1* and *BLMR2*, that were identified in Surpass 400-derived cultivars are thought to be either alleles of *LepR3* or to be masked by it [73].

*P. lingam* avirulence (*AvrLm*) genes interacting with these resistance genes have also been identified, but, in contrast with the latter, *AvrLm1*, *AvrLm2*, *AvrLm3*, *AvrLm4*, *AvrLm5*, *AvrLm6*, *AvrLm7*, *AvrLm9*, *AvrLm10*, *AvrLm11*, and *AvrLm14* have been cloned [74,75,76,77,78,79,80]. In North America, a study conducted in Manitoba, Canada, between 2010 and 2015 found the prevalence of the *P. lingam* avirulence genes *AvrLm2*, *AvrLm4*, *AvrLm5*, *AvrLm6*, *AvrLm7*, *AvrLm11*, and *AvrLmS* to be high, while those of the alleles *AvrLm1*, *AvrLm3*, *AvrLm9*, *AvrLepR1*, and *AvrLepR2* were low [81]; in the US, a recent study indicated that the genes *AvrLm2*, *AvrLm3*, *AvrLm5*, *AvrLm6*, and *AvrLm10* were at a high frequency, while the genes *AvrLm4-7*, *AvrLm9*, and *AvrLm11* were at moderate frequencies (del Rio, unpublished data); in this study, the presence of *AvrLep* genes was not investigated.

Temperature has been linked to differential responses in the protective activity of resistance genes in many pathosystems. For example, in canola, Noel et al. [82] indicated that *Rlm7* is temperature-sensitive and is effective at ≤20 °C (68 °F) but not at 25 °C (77 °F); they also concluded that *Rlm4* and *LepR3* are temperature-insensitive. Another example of interaction occurs between *Pseudomonas syringae* and *Arabidopsis thaliana*, where high temperatures (23–32 °C/73–90 °F) favor the PAMP-triggered immunity functions optimally, while at lower temperatures (10–23 °C/50–73 °F) effector-triggered immunity is more effective [83]. And in tobacco, the N gene is the best example of a temperature-sensitive R gene [84]. The effect of temperature was confirmed by Yang [85], who noted that larger hypersensitive lesions were produced at warmer temperatures. Not all R genes are affected by temperature; some remain stable across various temperatures. For example, *Sr21* offers stable resistance against the *Ug99* race of (Pgt) *Puccinia graminis tritici* [86]. In the case of canola crops, research was mainly focused on identifying the crucial months that influence the severity of *P. lingam* infections and lead to critical periods of disease management [87]. To mitigate these effects, developing and deploying resistant canola varieties carrying temperature-insensitive blackleg resistance genes is essential and may be possible through advanced breeding techniques such as marker-assisted selection and genomic selection. These techniques are invaluable in identifying and combining (R) genes from different brassica species [88]. Monitoring the pathogen population in changing climatic conditions along with developing resistant varieties is crucial for managing blackleg. Integration of these resistant varieties with other integrated disease management strategies can enhance their effectiveness and sustainability [89].

The risk posed by the durability of blackleg resistance in canola is increasing due to global climatic changes that result in pathogen adaptation and breakdown of major resistance genes under different climatic conditions. The only way to address this emerging threat is through canola breeding programs using different strategies, including stacking/pyramiding multiple resistance genes and incorporating quantitative resistance, and practices that reduce the selection pressure. Combining advanced genetic tools like CRISPR, genetic analyses like Genome-Wide Association Studies, and procedures like marker-assisted selection with traditional breeding methods may help accelerate the production of longer-lasting, climate-resilient resistant canola cultivars. For example, Wang et al. [90] introduced seven elite alleles for resistance to clubroot and tolerance to sulfonylurea herbicides into a male-fertility restorer line in *Brassica napus*. This line was used to produce six introgression lines to produce hybrids with strong resistance to clubroot and sulfonylurea herbicide. Lin et al. [91] used the former method to study the function of four homologous alleles of *BnaA07.MKK9* in response to infection by *S. sclerotiorum*. Pyramiding or stacking qualitative resistance genes alone is not the final solution, as their effectiveness can be lost when they are exposed repeatedly to recombinant populations of the pathogen [91]. *L. maculans* can produce such populations, and thus it possesses a high risk of overcoming the resistance genes. Evidence of the latter was provided by a study in France that revealed an increase in the prevalence of strains capable of infecting cultivars that carried the resistance genes *Rlm3* and *Rlm7* [92]. The pathogen population’s ability to change to a more virulent form in response to increased selection pressure also works in the reverse direction. Cultivars with defeated resistance genes can be redeployed in an area after monitoring activities indicate that the prevalence of their corresponding avirulence genes has increased sufficiently [93]. Combining qualitative and quantitative resistance genes may be another effective way to reduce selection pressure, as demonstrated by a five-year field study using near-isogenic lines of *Brassica napus* carrying the major resistance gene *Rlm6* and quantitative resistance genes [31]. While these examples reveal the potential of combining modern tools with traditional breeding methods, under climate-induced stress scenarios the effectiveness of these strategies remains largely underexplored. Future studies should prioritize evaluating resistance stability under variable temperature regimes. Integrating field-based assessments with molecular tools is essential to develop climate-smart, durable canola cultivars.

## 6. Cultural Practices (Crop Rotation and Residue Management) for Disease Management

Rotation of crops has played a significant role in reducing interactions between pathogens and crops. For example, a field study conducted from 1999 to 2002 in Manitoba revealed that planting canola every other year or at two-year intervals reduced disease severity compared to planting canola every year [36]; in contrast, under southern Australian conditions, blackleg was still present, albeit at moderate levels, in fields two years after the last canola crop but was almost absent by year three [94]. It is evident that warmer years resulting from climate change favor earlier pseudothecia maturation and loss of the protective activity of temperature-sensitive blackleg resistance genes. However, the increased temperatures and precipitation may speed up the degradation of crop residues where pseudothecia are formed. Nevertheless, rotating with a different crop for two to three years between canola plantings may help ameliorate disease pressure created by warmer years [95].

With the identification and cloning of avirulence genes, it is easier to detect the presence of effective resistance genes in commercial cultivars. This information has been successfully used in Australia [96] and Canada [97] for the strategic deployment of resistance genes to ease selection pressures and avoid blackleg outbreaks [98]. IDM strategies are cost-effective and environmentally friendly. Table 1 summarizes key examples proven to enhance yield and decrease blackleg incidence.

## 7. Chemical Control Methods

The lack of effective and high-level resistance in commercial canola cultivars led to the adoption of chemical control measures [102]. Typically, fungicides are applied as seed treatments, foliar spray, and coated fertilizer granules to manage blackleg of canola [103,104]. For example, *P. lingam* infections that occur after the five-leaf growth usually do not lead to yield-reducing stem cankers [105,106]; however, depending on environmental conditions, the protective activity of the seed treatment may be performed before the canola plants grow beyond this growth stage. Conversely, under more conducive conditions for plant development, seed treatments may provide longer and more effective protection. For example, in Australia, canola seeds treated with fluquinconazole were protected for up to six weeks from blackleg [107,108]. To extend protection and maximize yield, foliar fungicide applications between the second and fourth leaf growth stages is often recommended [108]. Delivery of fungicides like flutriafol as coatings for fertilizer grains can also improve control of blackleg [109]; but the extent of protection is short-lived, similar to seed treatments [110]. With the development of resistant cultivars, the economic return of a fungicide application depends on whether the protection offered by the resistance genes is sufficient to keep the disease at bay. For example, a positive return might occur when plants from a cultivar carrying *Rlm2* are sprayed because of the prevalence of blackleg strains in a particular field carrying avirulence genes other than *AvrLm2*. Conversely, cultivars carrying resistance genes that interact with similar avirulence genes may not see a positive return on the investment [105].

The availability and registration of fungicides and seed treatments highlight the significance of chemical control options as a component of integrated disease management strategies to combat blackleg disease effectively [43]. Three major classes of chemical fungicides are commonly used globally, including demethylation inhibitors (DMIs), quinone outside inhibitors (QoIs), and succinate dehydrogenase inhibitors (SDHIs) [111,112]. Eight seed treatment fungicides in North Dakota have been registered for use against blackleg disease. These fungicides are Dynasty (Syngenta, Basel, Switzerland), Rancona V RS (UPL Ltd., Mumbai, India), Prosper EverGol (Bayer CropScience, Monheim am Rhein, Germany), Maxim 4FS (Syngenta, Basel, Switzerland), Allegiance FL (Bayer CropScience, Monheim am Rhein, Germany), Helix Vibrance (Syngenta, Basel, Switzerland), Thiram 480 DP (Adama Ltd., Airport City, Israel), and Obvius (BASF SE, Ludwigshafen, Germany) were sourced and approved for use in field trials conducted in North Dakota, USA. Fungicides with different active ingredients have been used in different parts of the world to control blackleg disease of canola. For example, propiconazole has been commonly used as a foliar spray against blackleg in Canada, but in Europe, difenoconazole, flusilazole, and carbendazim have been in use since the early 1900s [31]. In the early 2000s, the QoI fungicides azoxystrobin and pyraclostrobin were introduced in Canada [110,113]. In most canola-growing regions of the United States, registered fungicides for early control of blackleg include azoxystrobin (Dynasty^®^), fluxapyroxad + pyraclostrobin (Obvius^®^), and fludioxonil (Maxim^®^) [114].

**Figure 7:**
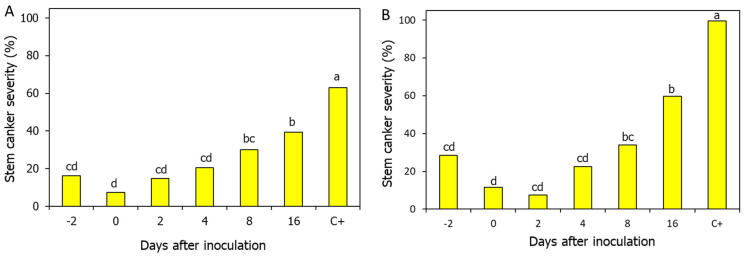
Effects of timing of (**A**) azoxystrobin and (**B**) pyraclostrobin applications (6 fl oz/A) in relation to the time of inoculation of seedlings of canola cv. Westar with *P. lingam* on the severity of blackleg stem canker in adult canola plants. Plants were sprayed with the fungicides 2 days prior, on the same day, or 2, 4, 8, or 16 days after inoculation. Non-sprayed plants (C+) were used as controls. Bars followed by the same letters are not significantly different, while different letters indicate statistically significant differences in disease severity among treatments.

Fungicide applications to foliage are recommended when canola plants are at the two to four leaf growth stages to ensure that the fungicide can prevent infections at the early stage of the crop. This recommendation is corroborated by the findings that fungicide applications made more than eight days after the inoculation of plants with the pathogen resulted in diminished protection (Figure 7) [32,46].

The use of single-site active pesticides always creates the possibility of resistance development in the target population. The blackleg–canola pathosystem is no exception. The resistance of *P. lingam* to DMI fungicides has been reported in Australia [115,116].

## 8. Biological Control Agents Used in Blackleg Management

Leveraging natural antagonists to suppress *P. lingam* offers an alternative approach to chemical fungicides [117]. While different types of microorganisms, including fungi and bacteria, have been identified for their biocontrol potential [118,119], very few commercial products have been registered for canola against blackleg. One such product, Serenade ASO, that contains the active strain *Bacillus subtilis* was withdrawn from the market less than a decade after its introduction. The lack of biological alternatives to manage blackleg in canola highlights the need for applied research in this area.

Another form of biological control is achieved when infection by a less aggressive parasite activates plant defenses, making the plant more resistant to subsequent infections by a more aggressive strain. This phenomenon is known as acquired systemic resistance (SAR) and has been observed when canola plants are infected by strains of *L. biglobosa* before being challenged with *P. lingam* [120,121]. Once activated in the host plant, the resistance is expressed both locally at the infection site and systemically throughout the plant. While the exact mechanisms through which SAR is elicited by *L. biglobosa* are still not understood, it has been shown that *L. biglobosa* strains infected with a double-stranded RNA quadri virus elicit a stronger SAR than non-infected strains [122]. SAR also can be elicited through the application of chemicals like benzo-(1,2,3)-thiadiazole-7-carbothioic acid S-methyl ester and BTH, which results in the accumulation of pathogenicity-related proteins and salicylic acid [123]. While biological control holds promise for managing blackleg in canola, its applicability remains limited. For example, identifying reliable microbial antagonists is difficult because *P. lingam* enters canola tissues quickly, making it hard for biocontrol agents to reach or survive at the infection court. To advance biocontrol, future research should focus on using microbial consortia that interact with the pathogens when they still are on infected residues as pycnidia or pseudothecia, as well as on plant endophytes, and rhizosphere manipulation, creating formulations for fields to integrate smoothly with current farming practices.

## 9. Challenges and Limitations of Current IDM Strategies

Several challenges and limitations hinder the effectiveness of integrated disease management strategies, including the development of resistance against commonly used fungicides, the breakdown of host resistance by pathogens, and the economic and practical feasibility of implementing diverse crop rotations and other cultural practices [124]. The development of resistance in pathogens against fungicides poses a significant threat to the sustainability of crop production systems [125]. This phenomenon can be caused by multiple factors, such as unnecessary applications of the same compound or of compounds with similar modes of action during the same growing season and the use of sublethal or excessively high doses [126]. Similar to many other host–pathogen interactions, management of blackleg disease in canola requires focusing on the above-mentioned limitations. For example, in Australia, a study showed that *P. lingam* had developed resistance to the triazole fungicide fluquinconazole, an otherwise very effective seed treatment. This situation increased the risk of occurrence of blackleg epidemics and created the need for additional management measures. Mutations in the *ERG11* gene, also known as *CYP51*, were responsible for the resistance development [127,128]. Fungicides are a valuable IDM tool but must be integrated with other cultural and genetic management strategies to ensure long-term effectiveness and sustainability. In Australia, the blackleg pathogen has shown resistance to various fungicides, leading to significant crop losses and necessitating changes in management strategies [115].

These measures include rotating fungicides with different modes of action and incorporating cultural practices to manage disease pressure [129]. Continued research into unraveling resistance mechanisms and developing integrated strategies will be crucial for maintaining effective control of blackleg disease in canola. In addition, excessive reliance on pesticides can expose humans to a variety of adverse health impacts, both immediate and long-term, that can significantly diminish a person’s overall well-being and quality of life [130,131]. There have been growing concerns over the role of climate change in exacerbating pesticide-related health risks in recent years as evidence indicates that rising temperatures associated with climate change may lead to an increase in the populations of certain pests [132,133,134,135]. In addition to promoting pest population growth, warmer temperatures can accelerate the breakdown of pesticides through volatilization and photodegradation [136,137].

## 10. Innovative IDM Approaches for Mitigating Climatic Changes

**Figure 8:**
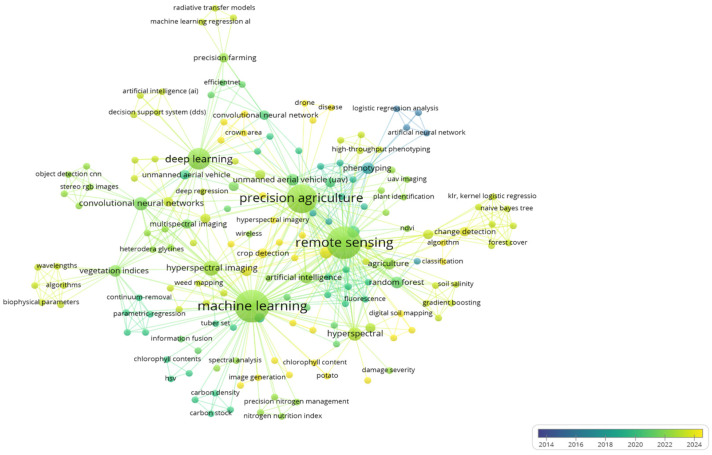
Bibliometric analysis of advanced tools currently used in agriculture.

Prompt detection of disease outbreaks is crucial for initiating timely and effective interventions, ultimately minimizing the impact on crops and global food security [138]. The ongoing evolution of the climate has intensified the challenges posed by plant diseases, threatening agricultural productivity and worsening conditions already conducive to disease outbreaks [139]. For instance, critical research focused on simulation models can accurately quantify the detrimental effects of plant pests and associated diseases on crop yield, enabling the development of timely and effective intervention strategies for plant disease forecasting [140]. An integrated disease management strategy with a comprehensive understanding of entire cropping ecosystems can be helpful in keeping plant diseases below the economic threshold level [141]. A robust decision-making process with a thorough understanding of disease etiology, epidemiology, cost–benefit analysis, and in-depth knowledge of plant protection measures are the cores of any IDM/IPM module [142,143]. IDM involves a significant decision-making process compared to conventional agricultural practices [144]. Predictive systems with formalized algorithms that assess various disease risk factors play an important role in facilitating informed decision making to determine the necessity of crop protection measures for integrated disease management [145]. Currently, many novel tools, including remote sensing [146], advanced predictive models, machine learning [147], precision agriculture, and decision support systems [148], empower growers to make more informed, data-driven decisions that lead to improved disease control and enhanced crop yield [149,150]. A detailed bibliometric analysis of available tools used in agriculture is given in Figure 8. Data was collected from PubMed and then analyzed using VOSviewer. Four clusters emerged after analysis: deep learning, remote sensing, machine learning, and precision agriculture. The co-occurrence of precision agriculture and remote sensing reflects the importance of monitoring crop health and environmental factors using real-time data. Similarly, the link between machine learning and precision agriculture indicates advancements in predictive analytics for crop management.

For instance, drones and satellites equipped with hyperspectral sensors or multispectral sensors can detect and capture high-resolution images of early disease stress across a canola field [151,152]. Some studies suggest that using hyperspectral imaging to detect pathogens in oilseed brassica [152] in conjunction with early disease detection systems could result in better management and ultimately reduce yield losses. Unmanned aerial vehicles equipped with hyperspectral imaging systems for early detection of diseases such as blackleg and Sclerotinia stem rot signal further refinements in technology that will be a significant component of IDM in the future [153]. Enhanced monitoring capability will result in more accurate identification of diseases that can occur in fields and thus facilitate the early implementation of management practices.

**Figure 9:**
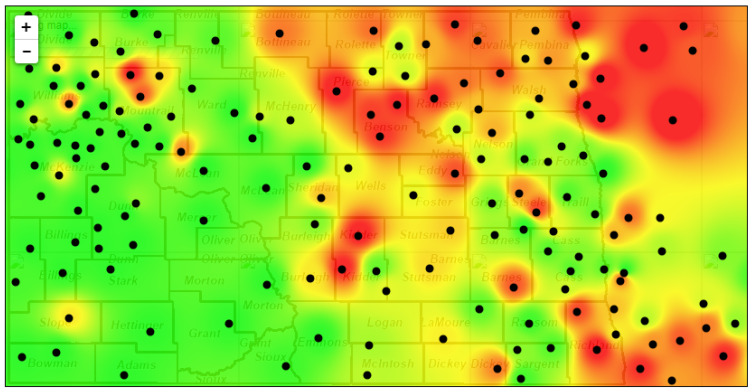
Risk map for Sclerotinia stem rot of canola. Areas in green depict a low risk of disease development, while yellow and red areas depict intermediate and high risks, respectively. This map is part of a disease warning system managed by the canola pathology program at North Dakota State University. Black dots represent weather stations from which data were collected for the algorithm that calculated risk.

Machine learning and artificial intelligence have revolutionized agriculture to help control different diseases of crops by analyzing large amounts of data from various sources [154]. Machine learning models can predict disease outbreaks and recommend optimal control strategies by integrating data from weather stations, soil sensors, and historical disease records [155]. Disease forecasting models specific to canola utilize environmental data, growth stages of crops, and pathogen race structures to predict the risk of blackleg development, helping farmers choose appropriate management strategies [156]. Another example of a blackleg forecasting model is Poland’s System for Forecasting Disease Epidemics (SPEC) [157]. A forecasting model has also been developed for Sclerotinia stem rot to help growers assess the risk of disease development and determine the need for fungicide application (Figure 9) [158]. The Sclerotinia model, which produces color-coded “risk maps” to inform farmers of the estimated risks, illustrates how spatial forecasting tools can support integrated disease management (IDM); similar geospatial models could be developed for blackleg of canola to support IDM and make timely decisions, especially as climate conditions continue to change.

Digitalized tools, including mobile applications and online platforms, are convenient resources that can be used to access disease risk maps, weather forecasts, and management recommendations for different canola diseases, including blackleg and stem rot [159]. In North Dakota, analyses using logistic regression have shown a link between the maturation of pseudothecia and climatic factors such as air temperature, measured in cumulative heat units, and relative humidity, quantified by the number of hours when the RH exceeds 75% (40). These analyses also provided evidence that pseudothecia appeared sooner and reached maturity at a notably quicker rate when the temperature was at 18 °C, as opposed to cooler conditions in North Dakota.

## 11. Conclusions and Future Directions

*Plenodomus lingam* poses a significant threat to canola production regions around the world. Diverse temperature effects and altered precipitation patterns due to climate change may exacerbate the impact of blackleg severely. Integrated management strategies are among the possible ways of mitigating the impact of climate change. IDM strategies should be focused on creating better forecasting models, the development of varieties that carry resistance genes that are not temperature-sensitive, improved fungicide compounds (more effective chemicals, RNA-based fungicides, and more efficient biocontrol agents), and more innovative crop rotation schemes that include rotation of resistance genes to assist canola farmers in determining the optimal timing for blackleg control. Emerging technologies such as AI-driven forecasting systems, precision agriculture, and genomic tools like CRISPR offer precision to strengthen IDM frameworks. To ensure practical adoption, interdisciplinary collaboration among pathologists, breeders, agronomists, and climate scientists is essential. Moreover, addressing socio-economic barriers and policy support mechanisms will be important in translating scientific innovations into sustainable solutions.

## Figures and Tables

**Table 1:** Key studies on synergic effects of IDM strategies [89,99,100,101].

Author	Title	Key Strategies Studied	Main Findings
Kutcher et al., 2013 [89]	Blackleg disease of canola mitigated by resistant cultivars and four-year crop rotations in western Canada	Crop rotation (2 to 4 years) + resistant cultivars (*Rlm3*, *RlmS*)	4-year rotation + resistant cultivars showed synergistic reduction in blackleg severity
Marcroft et al., 2012 [99]	Effect of rotation of canola cultivars with different complements of blackleg resistance genes on disease severity	Rotation of R genes (*Rlm1*, *Rlm4*, *Rlm6*) in different cultivars	Rotating resistance genes reduced selection pressure and delayed pathogen adaptation
Sprague et al., 2006 [100]	Major gene resistance to blackleg in Brassica napus is overcome by changes in virulence of populations of *Leptosphaeria maculans*	Monitoring resistance breakdown due to widespread use of R genes (*Rlm1*, *Rlm7*)	Field-level R gene breakdown observed; highlighted the need for integrated strategies
Crété et al., 2020 [101]	Rotating and stacking genes can improve crop resistance durability	Modeling of gene rotation, pyramiding, and mixtures	Rotation outperformed pyramiding in maintaining resistance durability against recombining pathogens

## Data Availability

No new data were created or analyzed in this study. Data sharing is not applicable to this article.
